# Foliar trehalose pretreatment alleviates physiological and metabolic damage of ‘Kyoho’ grape under low-temperature stress

**DOI:** 10.3389/fpls.2026.1982336

**Published:** 2026-10-28

**Authors:** Yanyu Ma, Jiayin Niu, Qian Yu, Yihe Yu

**Affiliations:** 1 College of Horticulture and Plant Protection, Henan University of Science and Technology, Luoyang, China; 2 Henan Provincial Engineering Research Center on Characteristic Berry Germplasm Innovation and Utilization, Luoyang, China; 3 Faculty of Business and Trade, Wuhan Institute of Shipbuilding Technology, Wuhan, China

**Keywords:** concentration effect, exogenous trehalose, ‘Kyoho’ grape, low-temperature stress, physiological mechanism

## Abstract

**Background:**

Low temperature is a major abiotic stress that limits grape production in cooler regions. The cell membrane is the primary target of low-temperature injury, and the membrane lipid peroxidation chain reaction triggered by a burst of reactive oxygen species (ROS) drives the loss of membrane integrity and the rise in permeability. Foliar application of exogenous regulators such as trehalose is an effective measure to enhance plant cold resistance; however, the concentration-dependent effects of exogenous trehalose on grape cold resistance remain unclear.

**Methods:**

One-year-old ‘Kyoho’ grape cutting seedlings were foliar-sprayed with trehalose at 50–250 mmol L^−1^ and, 24 h later, exposed to 0 °C for 7 d. Membrane integrity, reactive oxygen species, antioxidant enzymes and non-enzymatic antioxidants, osmotic adjustment substances, ion balance, and energy-respiratory metabolism indicators were measured, and principal component analysis (PCA) was performed.

**Results:**

Compared with the unstressed control (CK), low-temperature stress severely damaged leaf cell membranes, markedly increasing relative electrolyte conductivity (REC) and malondialdehyde (MDA) content (p < 0.05), and disrupted intracellular ROS homeostasis, with significant elevations of hydrogen peroxide (H_2_O_2_) and superoxide anion (O2^·−^). Exogenous trehalose mitigated ROS accumulation and membrane lipid peroxidation in a hormesis-like, concentration-dependent manner: protection increased from T1 (50 mmol L^−1^) to a peak at T2 (100 mmol L^−1^)—which significantly reduced REC, MDA, H_2_O_2_ and O2^·−^ relative to the low-temperature (LT) group (p < 0.05)—and declined at T3–T5, being largely lost at 250 mmol L^−1^. The optimal 100 mmol L^−1^ treatment also enhanced enzymatic (SOD, POD, CAT) and non-enzymatic (AsA, GSH, soluble sugars, soluble protein, total phenols) antioxidant defenses, promoted energy-respiratory metabolism (CCO, NAD(H), NADP(H), respiration rate) and osmotic adjustment (soluble sugars, soluble protein, proline, glycine betaine), and maintained Na^+^/K^+^ balance. PCA separated the 100 mmol L^−1^ group from LT, closest to CK, with the first two principal components explaining 68.9% of the total variance.

**Conclusion:**

These findings indicate that an appropriate concentration of exogenous trehalose partially preserves membrane integrity and alleviates oxidative injury under low-temperature stress through coordinated regulation of antioxidant defense and energy-respiratory metabolism, providing a quantitative basis for the application of exogenous trehalose in cold-resistant grape cultivation.

## 1 Introduction

Grape (*Vitis* spp.) is a high-value fruit crop cultivated worldwide for fresh consumption, winemaking and processing, and it occupies a central position in the global fruit industry and agricultural economy (Chaves et al., 2003; Amulya et al., 2024). Among the various abiotic stresses that affect grapevines throughout the growing season, low temperature is a major factor limiting cultivation range, inhibiting growth, and reducing yield and quality (Chen et al., 2022; Zhang et al., 2023). Under global climate change, extreme low-temperature events such as late-spring and early-autumn frosts are becoming more frequent; these readily cause growth stagnation and a sharp decline in photosynthetic efficiency, and in severe cases shoot wilting and flower-bud necrosis, resulting in substantial economic losses to the grape industry (Chen et al., 2022; Zhang et al., 2023).

Low temperature inflicts systemic damage on grapevines. It directly disrupts cellular homeostasis and induces massive accumulation of reactive oxygen species (ROS) such as superoxide anion and hydrogen peroxide, thereby triggering membrane lipid peroxidation and respiratory dysfunction (Hasanuzzaman et al., 2020). Low temperature also destabilizes photosynthetic electron transport in chloroplasts, lowers the photochemical efficiency of photosystem II (PSII), and may cause photoinhibition or even irreversible damage to the photosynthetic apparatus (Takahashi and Murata, 2008). In addition, it disturbs cellular water and ion homeostasis, hinders normal growth and development, and ultimately constrains yield and quality formation (Walker et al., 2000; Aran et al., 2017). ‘Kyoho’ (*Vitis vinifera* × *Vitis labrusca* ‘Kyoho’) is a leading fresh-eating hybrid cultivar widely planted in China, but its weak cold tolerance makes it prone to photosynthetic decline and metabolic disorder under low temperature, which greatly limits its cultivation in cooler regions (Ren et al., 2023; Zhang et al., 2023). Elucidating the low-temperature response of ‘Kyoho’ grape and developing efficient, environment-friendly measures to enhance its stress resistance are therefore of considerable theoretical and practical value for improving cultivation systems in cold-risk areas and strengthening the resilience of the grape industry.

During evolution, plants have acquired a coordinated cold-resistance system in which osmotic adjustment, antioxidant defense and energy-respiratory metabolism act in concert to limit low-temperature damage (Hasanuzzaman et al., 2020; Chaves et al., 2003). Osmotic adjustment relies on small molecules such as soluble sugars to sustain intracellular osmotic pressure and reduce freezing injury (Bates et al., 1973); the antioxidant system scavenges excess ROS through the combined action of enzymatic and non-enzymatic constituents, thereby alleviating membrane lipid peroxidation (Foyer and Halliwell, 1976; Nakano and Asada, 1981; Rahman et al., 2006); and energy-respiratory metabolism, a central component of stress resistance, sustains the energy supply by stabilizing energy charge and regulating key respiratory enzymes (Ribas-Carbo et al., 1994). Together, these coordinated mechanisms provide a theoretical basis for the present study.

Trehalose is a non-reducing disaccharide that functions both as an osmolyte and as a signal molecule, and it has considerable potential for improving plant abiotic-stress tolerance (Shi et al., 2019; Raza et al., 2024). Enhanced trehalose metabolism or exogenous application has been reported to improve the stress tolerance of crops such as rice (Li et al., 2011) and watermelon (Yuan et al., 2022) by activating the antioxidant system and stabilizing the photosynthetic apparatus, and it also alleviates abiotic-stress damage in fruit and vegetable crops through antioxidant and metabolic regulation (Feng et al., 2019; Yuan et al., 2022). In fruit trees, exogenous regulators show clear concentration-dependent effects on stress resistance, and energy-respiratory metabolism is a core pathway of the grapevine stress response (Yu et al., 2021). The protective effect of trehalose is, however, concentration-dependent and species-specific: for gramineous crops such as wheat and rice, foliar application at 10–50 mmol L^−1^ has generally proved effective, whereas excessive concentrations aggravate damage (Sadak, 2019; Soares et al., 2023). Although the suitable concentration range of trehalose for grape has been preliminarily explored, the optimal threshold and the associated energy-respiratory regulatory pathway under low-temperature stress have not been systematically defined for ‘Kyoho’ grape, and the mechanism by which trehalose alleviates low-temperature stress in grape remains less well characterized than that of regulators such as salicylic acid and glycine betaine. In this study, one-year-old ‘Kyoho’ cutting seedlings were foliar-sprayed with a trehalose concentration gradient (50–250 mmol L^−1^) and then subjected to 0 °C stress. Indicators covering membrane integrity, the antioxidant system, osmotic adjustment, ion homeostasis and energy-respiratory metabolism were systematically measured. The aims were to (i) determine the optimal concentration of exogenous trehalose for alleviating low-temperature stress in ‘Kyoho’ grape and (ii) characterize the underlying physiological mechanism, with emphasis on the coordinated regulation of energy-respiratory metabolism and antioxidant defense, so as to provide a scientific basis for chemical regulation of cold-resistance cultivation in grape-producing regions at risk of low temperature.

## 2 Materials and methods

### 2.1 Plant materials

One-year-old ‘Kyoho’ grape cutting seedlings that were uniform in growth and free of diseases, pests and mechanical damage were used as the experimental materials. The screening criteria were plant height of 30–35 cm, basal stem diameter of 0.5–0.6 cm, main root length of 10–15 cm, and fibrous root number ≥15, to ensure the uniformity of the plant material and minimize experimental error.

Seedlings were cultured following standard protocols for grapevine physiological studies (Zhang et al., 2023). The seedlings were transplanted into plastic nutrient pots with a diameter of 15 cm and a height of 20 cm, with one seedling per pot. The cultivation substrate was quartz sand (particle size 0.5–1.0 mm) disinfected with 0.1% potassium permanganate. All plants were cultured in an RXZ-500D artificial climate chamber under the following conditions: day temperature 25 ± 2 °C, night temperature 20 ± 2 °C, relative humidity 60 ± 5%, photosynthetically active radiation 300 μmol m^−^² s^−1^, and photoperiod 16 h/8 h. Half-strength Hoagland nutrient solution (pH 6.0–6.5) was irrigated daily at 09:00 to ensure normal nutrient supply for plants.

Trehalose (analytical grade, purity ≥99%, Shanghai Yuanye Bio-Technology Co., Ltd., Shanghai, China) and Tween-80 (Beijing Solarbio Science and Technology Co., Ltd., Beijing, China) were used. A physiological indicator kit for cytochrome c oxidase (CCO) was purchased from Beijing Solarbio Science and Technology Co., Ltd. All other reagents were of analytical grade.

### 2.2 Experimental methods

#### 2.2.1 Experimental design

Based on common protocols for low-temperature studies in grapevine and on our preliminary experiments, the stress temperature was set at 0 °C. This temperature reliably induces a cold-stress response without causing rapid plant death and clearly differentiates the cold tolerance of the different treatments (Aran et al., 2017; Chen et al., 2022; Zhang et al., 2023).

The experiment consisted of a normal-temperature control (CK), a low-temperature stress control (LT) and five trehalose treatments (T1-T5) at 50, 100, 150, 200 and 250 mmol L^−1^, respectively. The CK and LT plants were sprayed with distilled water containing 0.1% (v/v) Tween-80. Tween-80, a non-ionic surfactant, was included in every spray to reduce the surface tension of the solution on the waxy leaf cuticle and to ensure uniform adhesion and foliar uptake of trehalose; because the same concentration was applied to all groups, any surfactant-specific effect was balanced across treatments and does not confound the treatment comparisons. The trehalose concentrations were selected on the basis of previous studies and our preliminary experiments (Li et al., 2011; Yuan et al., 2022; Pan et al., 2023). After 15 d of seedling acclimation, foliar spraying was performed once at 09:00 at a volume of 10 mL per plant, ensuring that both leaf surfaces were wetted without run-off. Twenty-four hours after spraying, the LT group and all trehalose treatments were transferred to a climate chamber at 0 °C for 7 d of stress treatment, whereas the CK group was maintained under conventional culture conditions (Jia et al., 2023).

Sampling was performed between 09:00 and 10:00 on day 7 of the low-temperature treatment. The top 3–4 fully expanded functional leaves were collected from each plant, and three leaves were pooled per biological replicate. Samples were rapidly frozen in liquid nitrogen and stored at -80 °C until analysis. All sampling and storage procedures followed standard protocols for stress-physiology studies in fruit trees (Chang et al., 2005; Zhang et al., 2023). The experiment comprised three biological replicates, each consisting of 10 plants. The experimental design is summarized in **Table 1**, and a schematic flowchart of seedling acclimation, foliar spraying, low-temperature treatment and sampling was provided as **Supplementary Figures S1**, **S2**.

**Table 1:** Experimental design of exogenous trehalose regulating low-temperature stress in ‘Kyoho’ grape.

No.	Treatment name	Culture temperature	Foliar spraying treatment 24 h before low temperature treatment
1	Normal temperature control (CK)	25 ± 2 °C/20 ± 2 °C (day/night)	Distilled water containing 0.1% (v/v) Tween-80
2	Low temperature stress control (LT)	0 °C	Distilled water containing 0.1% (v/v) Tween-80
3	T1 treatment	0 °C	50 mmol L^−1^ trehalose solution containing 0.1% (v/v) Tween-80
4	T2 treatment	0 °C	100 mmol L^−1^ trehalose solution containing 0.1% (v/v) Tween-80
5	T3 treatment	0 °C	150 mmol L^−1^ trehalose solution containing 0.1% (v/v) Tween-80
6	T4 treatment	0 °C	200 mmol L^−1^ trehalose solution containing 0.1% (v/v) Tween-80
7	T5 treatment	0 °C	250 mmol L^−1^ trehalose solution containing 0.1% (v/v) Tween-80

All treatments were set up with 3 biological replicates, and each replicate contained 10 plants. Trehalose solutions were prepared with distilled water, and the trehalose used in the experiment was analytical grade (purity ≥99%, Shanghai Yuanye Bio-Technology Co., Ltd.).

#### 2.2.2 Determination of indicators and methods

Unless otherwise stated, all enzyme activities and metabolite contents are expressed on a fresh-weight (FW) basis. The exceptions are glycine betaine, Na^+^ and K^+^ contents, all of which are expressed on a dry-weight (DW) basis. No parameter was normalized to soluble protein. Units for each indicator are given below.

#### (1) Membrane system and antioxidant system indicators

relative electrolyte conductivity (REC): Determined by conductivity meter method. Leaf discs were soaked in distilled water, and the initial electrical conductivity and total electrical conductivity after boiling were measured. REC was calculated as the ratio of the initial to the total electrical conductivity: $REC=(\frac{Initial\;electrical\;conductivity}{Total\;electrical\;conductivity\;after\;boiling})\times 100$ (Pan et al., 2023).

Malondialdehyde (MDA) content: Determined by thiobarbituric acid (TBA) colorimetric method. The absorbance at 532 nm and 600 nm was measured to calculate the content, with units of nmol g^−1^ FW (Velikova et al., 2000).

Reactive oxygen species (ROS) content: Superoxide anion (O_2_·^−^) production rate was determined by the hydroxylamine oxidation method, and hydrogen peroxide (H_2_O_2_) content was determined by the titanium sulfate method, with μmol g⁻¹ FW h⁻¹ and μmol g⁻¹ FW, respectively (Velikova et al., 2000).

Antioxidant enzyme activities: Superoxide dismutase (SOD) activity was determined by nitroblue tetrazolium photochemical reduction method, peroxidase (POD) activity by guaiacol method, and catalase (CAT) activity by potassium permanganate titration method. All enzyme activities were expressed in U g^−1^ FW, and the determinations followed the classical methods of stress-resistance physiology (Nakano and Asada, 1981; Feng et al., 2019). Non-enzymatic antioxidant substances: AsA and GSH contents were determined following Kampfenkel et al. (1995) and Rahman et al. (2006). Total phenols and flavonoids were determined by the Folin–Ciocalteu and AlCl_3_ colorimetric methods, respectively, and the results were expressed on a fresh weight basis (AsA and GSH in μmol g^−1^ FW, and total phenols and flavonoids in mg g^−1^ FW) (Jia et al., 1999; Singleton et al., 1999).

#### (2) Osmotic adjustment and ion balance indicators

Osmotic adjustment substances: Soluble sugar content was determined by the anthrone colorimetric method, soluble protein content by the Coomassie brilliant blue G-250 dye-binding method, and glycine betaine content by the spectrophotometric periodide method (extracted with methanol-chloroform, precipitated as betaine periodide with potassium triiodide, and measured at 365 nm). Results were expressed on a fresh weight basis (soluble sugars and soluble proteins in mg g⁻¹ FW; glycine betaine in μg g⁻¹ DW) (Yang et al., 2012). Free proline (Pro) content: Determined by ninhydrin colorimetric method. Samples were extracted with 3% sulfosalicylic acid and then developed in a boiling water bath. The absorbance at 520 nm was measured to calculate the content, with the unit of μg g^−1^ FW (Bates et al., 1973).

Ion contents: Dried leaves were digested with concentrated sulfuric acid-hydrogen peroxide, and the contents of Na^+^ and K^+^ were determined using an ICS-3000 ion chromatograph and expressed on a dry weight (DW) basis to calculate the Na^+^/K^+^ ratio (Afzal et al., 2021).

#### (3) Energy and respiratory metabolism indicators

NAD(H) and NADP(H) contents: Determined according to the method of Gibon and Larher (1997). Samples were extracted with acidic and alkaline solutions in an ice bath respectively, and the contents were measured after color development, with the unit of nmol g⁻¹ FW.

Cytochrome c oxidase (CCO) activity: Crude mitochondria were extracted from leaves according to the method of Douce et al. (1987). The absorbance change was measured after constant temperature reaction at 37 °C. One unit (U) of enzyme activity was defined as the amount of enzyme that oxidizes 1 μg of cytochrome c per minute, and CCO activity was expressed as U g^−1^ FW (Cooperstein and Lazarow, 1951).

Respiration rate: Determined by infrared gas analysis. Fresh leaves were dark-adapted at 27 °C for 1 h, and dark respiration was then measured with an LI-6400XT portable photosynthesis system (LI-COR Biosciences, Lincoln, NE, USA) at 27 °C. Respiration rate was expressed as mg CO_2_ kg^−1^ FW h^−1^ (Ribas-Carbo et al., 1994).

### 2.3 Data statistics and analysis

Data were compiled in Excel 2021, and the results were expressed as mean ± standard deviation (Mean ± SD). One-way analysis of variance (ANOVA) was performed using SPSS 26.0 software, and Duncan’s multiple range test was used for multiple comparisons (p < 0.05). Pearson correlation analysis was conducted to analyze the correlation between indicators (p < 0.05, p < 0.01 or p < 0.001). Graphs were prepared with GraphPad Prism 10.1.2 and exported at 600 dpi in TIFF format. Individual biological replicates (n = 3) are shown as overlaid scatter points on each bar. Principal component analysis (PCA) was performed using SPSS 26.0 (IBM Corp., Armonk, NY, USA) on all measured physiological indicators (z-score standardized prior to analysis). Treatment groups (CK, LT, T1-T5) were projected onto the first two principal components, and 95% confidence ellipses were drawn for each group to visualize within-group dispersion and between-group separation.

## 3 Results

### 3.1 Effects of exogenous trehalose on cell membrane stability and reactive oxygen species accumulation in ‘Kyoho’ grape leaves under low-temperature stress

The cell membrane is the primary target of low-temperature stress in plants, and the membrane lipid peroxidation chain reaction triggered by a burst of reactive oxygen species (ROS) drives the loss of membrane integrity and the rise in permeability. As shown in **Figure 1**, compared with the unstressed control (CK), 0 °C low-temperature stress severely damaged the cell membranes of ‘Kyoho’ grape leaves, markedly increasing relative electrolyte conductivity (REC) and malondialdehyde (MDA) content (p < 0.05). Intracellular ROS homeostasis was simultaneously disrupted: hydrogen peroxide (H₂O₂) and superoxide anion (O₂·⁻) were both significantly elevated relative to CK (p < 0.05), indicating marked oxidative injury under low-temperature stress. Pretreatment with exogenous trehalose significantly mitigated the low-temperature-induced accumulation of ROS and membrane lipid peroxidation, and the protective effect followed a hormesis-like dose–response: it peaked at an intermediate concentration and waned at supra-optimal doses. From T1 (50 mmol L⁻¹) to T2 (100 mmol L⁻¹), REC, MDA content and ROS burden decreased progressively with rising trehalose concentration. T2 was the most effective treatment, producing significantly lower REC, MDA, H₂O₂ and O₂·⁻ than the low-temperature (LT) group (p < 0.05). Protection then declined at T3 (150 mmol L⁻¹) and further at T4 (200 mmol L⁻¹) and T5 (250 mmol L⁻¹): under T5, REC and O₂·⁻ did not differ significantly from the LT level, whereas MDA and H₂O₂ remained significantly lower than in LT. Together, these data indicate that an appropriate concentration of exogenous trehalose partially preserves membrane integrity and alleviates oxidative injury under low-temperature stress, consistent with efficient scavenging of stress-accumulated ROS and dampening of the membrane lipid peroxidationchain reaction.

**Figure 1:**
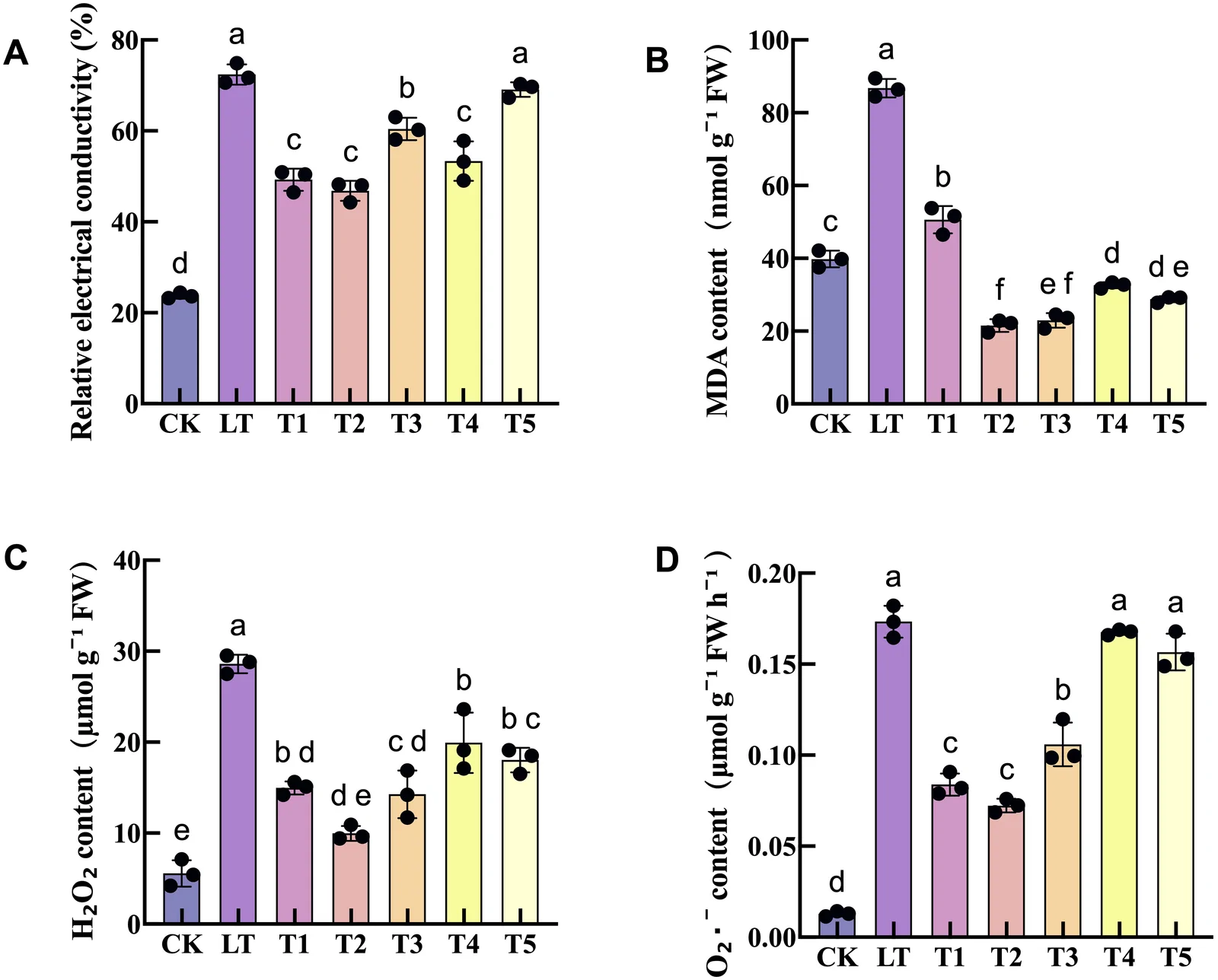
Effects of different concentrations of exogenous trehalose on cell membrane stability and ROS accumulation in ‘Kyoho’ grape leaves under low-temperature stress. **(A)** Relative electrolyte conductivity (REC, %). **(B)** Malondialdehyde (MDA, nmol g^−1^ FW). **(C)** Hydrogen peroxide (H_2_O_2_, μmol g^−1^ FW). **(D)** Superoxide anion production rate (O_2_·^−^, μmol g^−1^ FW h^−1^). Different lowercase letters indicate significant differences among treatments (Duncan’s multiple range test, p < 0.05). Error bars represent the mean ± standard deviation (Mean ± SD, n = 3).

### 3.2 Regulatory effects of exogenous trehalose on the antioxidant defense system under low-temperature stress

The antioxidant defense system is the core system for plants to scavenge excessive intracellular ROS and resist oxidative stress and comprises enzymatic and non-enzymatic antioxidant systems that act synergistically.

#### 3.2.1 Regulation of antioxidant enzyme activities

Superoxide dismutase (SOD), peroxidase (POD) and catalase (CAT) are key components of the plant enzymatic antioxidant system, and their synergistic action forms the first line of defense against ROS. As shown in **Figure 2**, compared with CK, 0 °C low-temperature stress significantly inhibited the activities of all three enzymes in ‘Kyoho’ grape leaves (p < 0.05): the activities of SOD, CAT and POD in the LT group were 28.6%, 38.0% and 32.2% lower than in CK, indicating that low-temperature stress directly compromised the enzymatic antioxidant defense and sharply reduced ROS-scavenging capacity.

**Figure 2:**
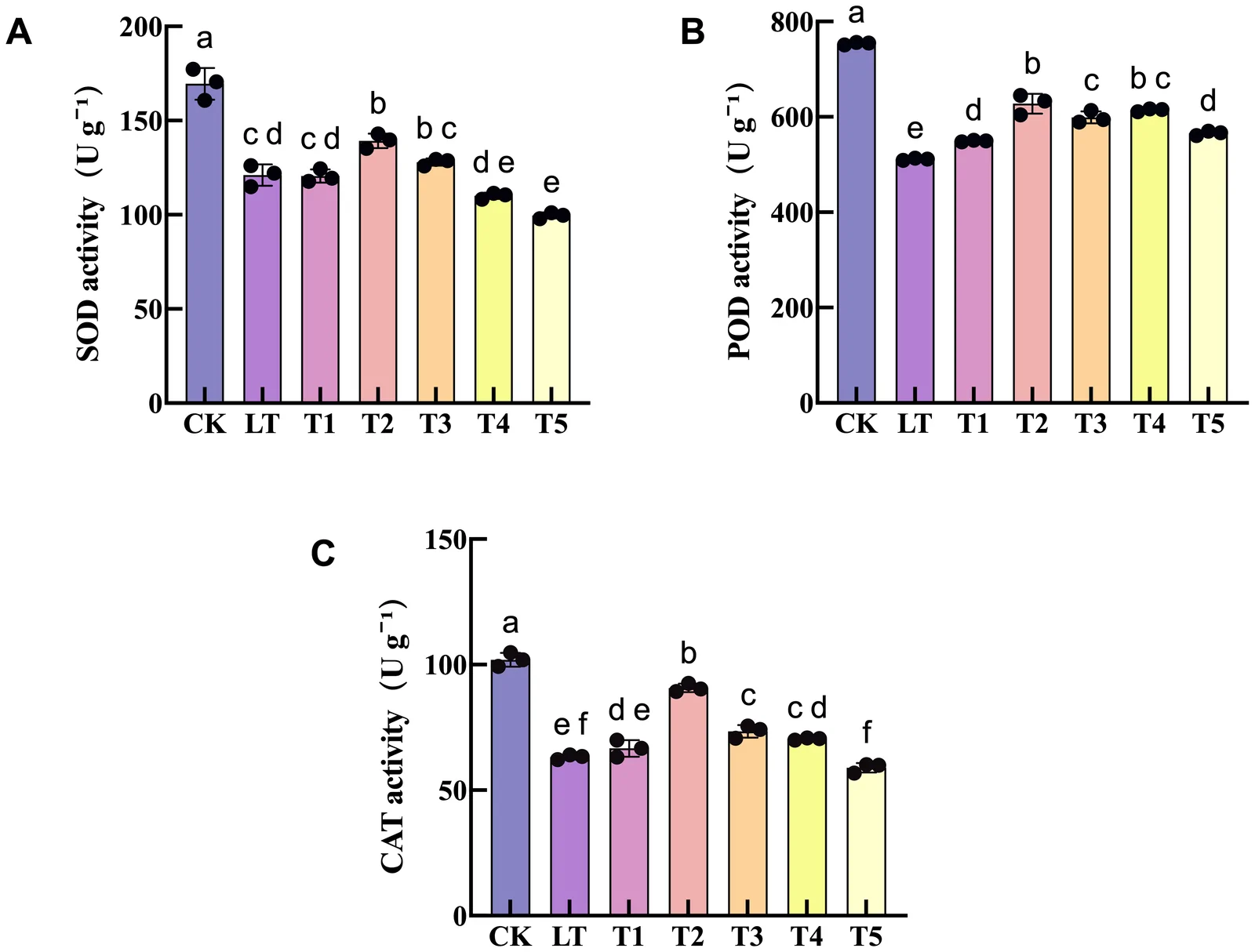
Effects of different concentrations of exogenous trehalose on antioxidant enzyme activities in ‘Kyoho’ grape leaves under low-temperature stress. **(A)** Superoxide dismutase (SOD, U g^−1^ FW). **(B)** Peroxidase (POD, U g^−1^ FW). **(C)** Catalase (CAT, U g^−1^ FW). Different lowercase letters indicate significant differences among treatments (Duncan’s multiple range test, p < 0.05). Error bars represent the mean ± standard deviation (Mean ± SD, n = 3).

Pretreatment with exogenous trehalose modulated the activities of these enzymes in a concentration-dependent manner, with the strongest overall activation under T2. Compared with the LT group, T2 significantly increased the activities of SOD, CAT and POD by 15.0%, 43.6% and 22.8%, respectively (p < 0.05). Among the other suboptimal treatments, T3 (150 mmol L^−1^) significantly enhanced POD and CAT activities and T1 (50 mmol L^−1^) significantly enhanced POD activity, whereas SOD activity was not significantly affected by either treatment. At the highest concentrations (T4 and T5), the three enzymes responded divergently: POD activity remained significantly higher than in the LT group even under T4 and T5, CAT activity was still significantly enhanced under T4 but did not differ significantly from the LT level under T5, and SOD activity was significantly inhibited under T5, whereas the decrease under T4 was not statistically significant. These results indicate that 100 mmol L^−1^ exogenous trehalose most effectively reinforces the enzymatic ROS-scavenging system, whereas supra-optimal concentrations disrupt the coordinated response of the three enzymes, particularly that of SOD. It should be noted that the present study measured enzyme activities rather than transcript levels or post-translational modifications; whether the observed changes in enzyme activity involve transcriptional regulation or post-translational modification therefore remains to be elucidated.

#### 3.2.2 Regulation of non-enzymatic antioxidant substance contents

Ascorbic acid (AsA), reduced glutathione (GSH), total phenols and flavonoids are core non-enzymatic antioxidants in plants, and the AsA-GSH cycle is the central pathway of non-enzymatic ROS scavenging. As shown in **Figure 3**, compared with CK, 0 °C low-temperature stress significantly decreased the contents of AsA and GSH in ‘Kyoho’ grape leaves (p < 0.05), whereas the contents of total phenols and flavonoids decreased numerically but not significantly, indicating that the non-enzymatic antioxidant defense system was also disturbed.

**Figure 3:**
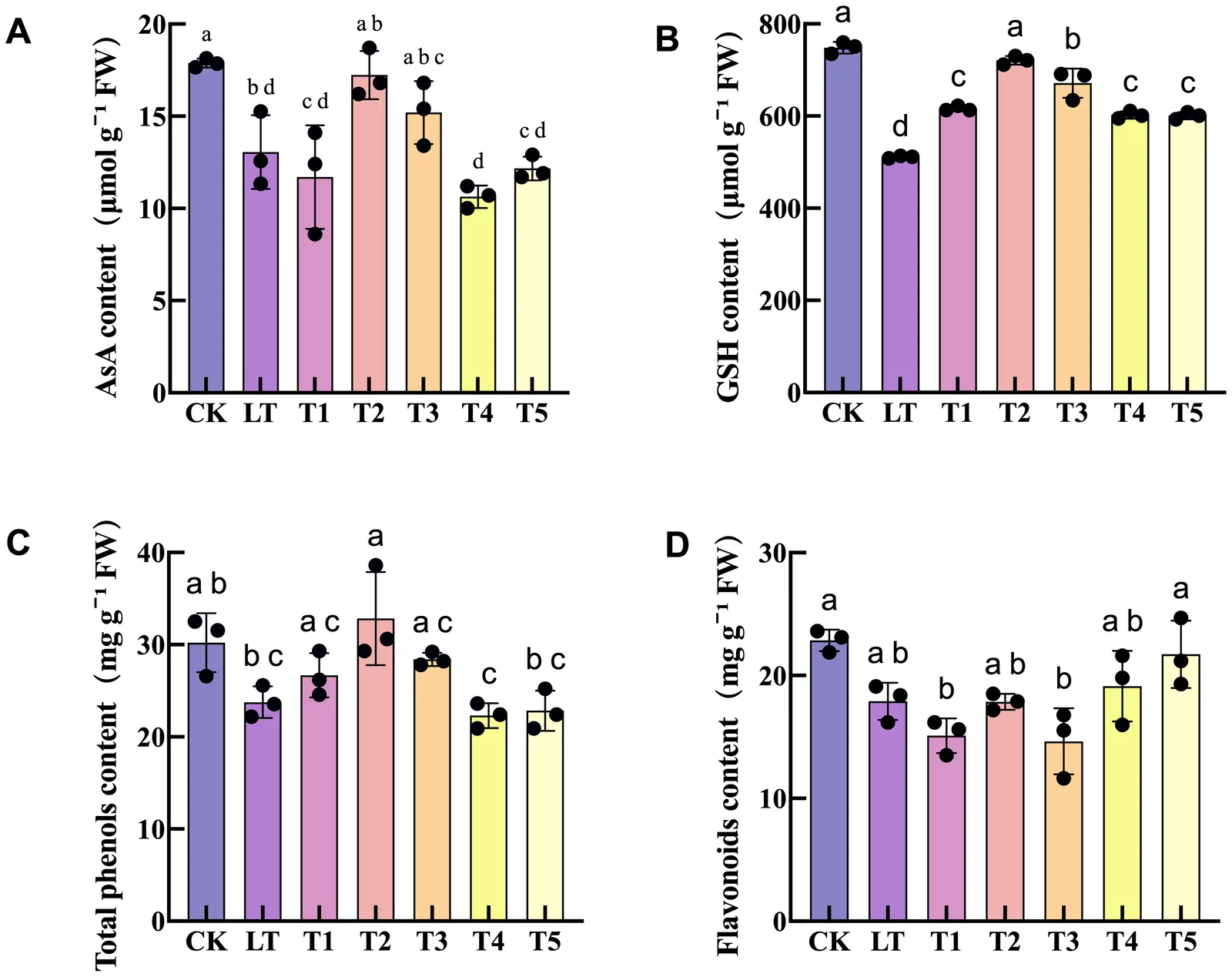
Effects of different concentrations of exogenous trehalose on non-enzymatic antioxidant substance contents in ‘Kyoho’ grape leaves under low-temperature stress. **(A)** Ascorbic acid (AsA, μmol g^−1^ FW). **(B)** Reduced glutathione (GSH, μmol g^−1^ FW). **(C)** Total phenols (mg g^−1^ FW, expressed as gallic acid equivalent). **(D)** Flavonoids (mg g^−1^ FW, expressed as rutin equivalent). Different lowercase letters indicate significant differences among treatments (Duncan’s multiple range test, p < 0.05). Error bars represent the mean ± standard deviation (Mean ± SD, n = 3).

Pretreatment with exogenous trehalose further regulated the accumulation of non-enzymatic antioxidants in a concentration-dependent manner. T2 showed the strongest promotion: compared with the LT group, the contents of GSH and total phenols increased significantly (p < 0.05), the AsA content was increased numerically (+32.0%) but did not reach statistical significance, and the flavonoid content was essentially unchanged. GSH responded most strongly to T2 among the non-enzymatic antioxidants, suggesting that the AsA-GSH cycle may be the core non-enzymatic pathway through which trehalose strengthens antioxidant defense under low temperature in grape. T3 restored the contents of AsA and total phenols to the CK level and also significantly increased the GSH content, while T1 significantly increased the GSH content; the flavonoid contents under T1 and T3, by contrast, did not differ significantly from LT. At the high concentrations (T4 and T5), the contents of AsA and total phenols no longer differed significantly from LT, although the GSH content remained significantly higher; the flavonoid content recovered to the CK level under T5. Taken together with Section 3.2.1, these results indicate that appropriate concentrations of exogenous trehalose comprehensively enhance the low-temperature antioxidant capacity of grape seedlings by acting on both the enzymatic and the non-enzymatic antioxidant defense systems.

### 3.3 Effects of exogenous trehalose on the accumulation of osmoprotectants under low-temperature stress

Osmotic adjustment is an important strategy by which plants tolerate low-temperature-induced cellular dehydration and freezing injury: by accumulating soluble osmoprotective solutes, plants lower their cellular osmotic potential, maintain turgor and water balance, and protect membrane and protein structures. As shown in **Figure 4**, the contents of soluble sugars and glycine betaine in ‘Kyoho’ grape leaves were significantly lower in the LT group than in the CK group (p < 0.05), whereas the soluble protein content decreased only slightly (not significantly), suggesting that low temperature weakened the osmotic adjustment capacity of the seedlings. Proline behaved differently: its content rose sharply under low temperature, indicating that proline was the main osmoprotectant synthesized in response to cold stress.

**Figure 4:**
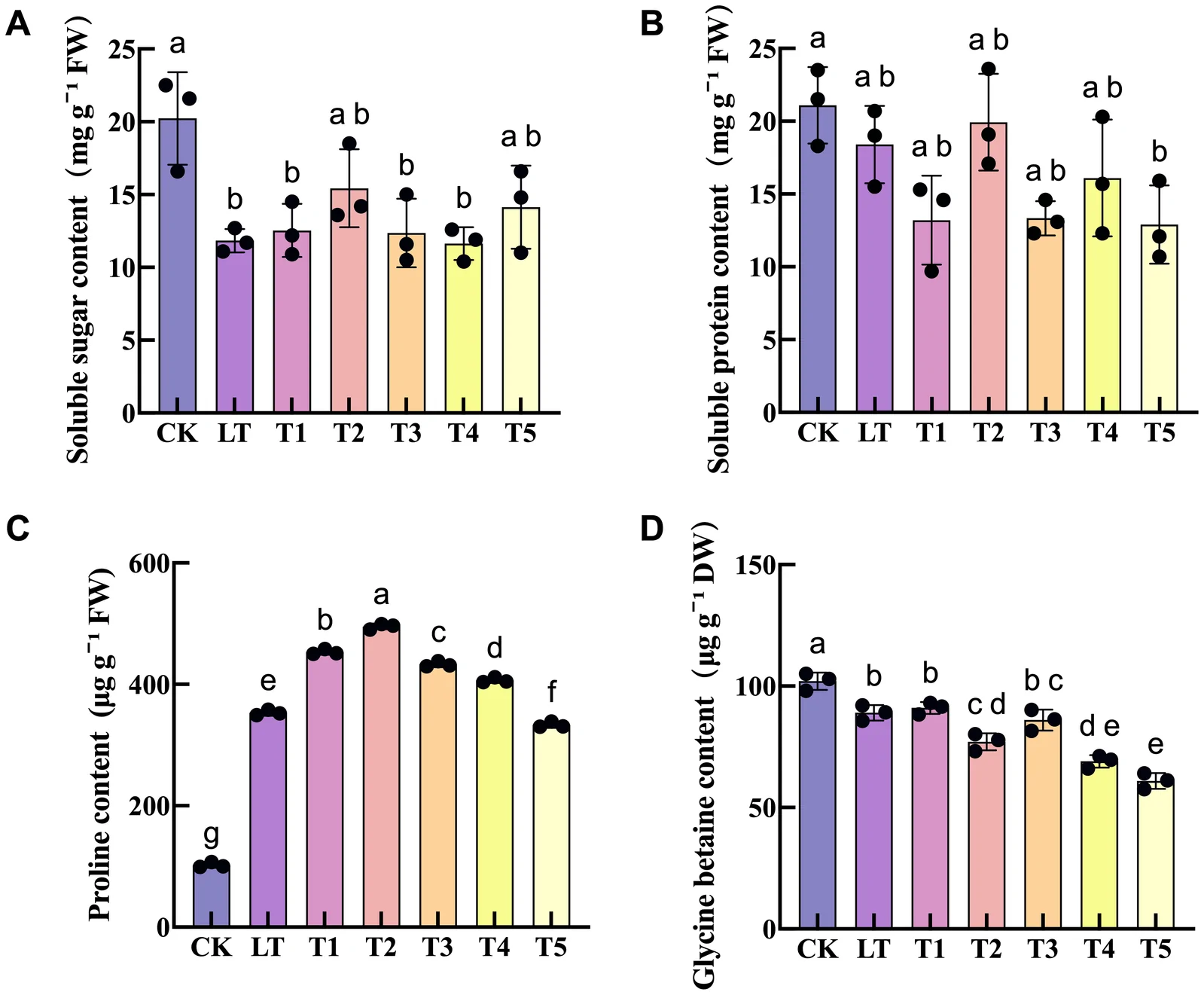
Effects of different concentrations of exogenous trehalose on osmoprotectant contents in ‘Kyoho’ grape leaves under low-temperature stress. **(A)** Soluble sugars (mg g^−1^ FW). **(B)** Soluble proteins (mg g^−1^ FW). **(C)** Proline (μg g^−1^ FW). **(D)** Glycine betaine (μg g⁻¹ DW). Different lowercase letters indicate significant differences among treatments (Duncan’s multiple range test, p < 0.05). Error bars represent the mean ± standard deviation (Mean ± SD, n = 3).

Trehalose pretreatment altered the accumulation of these solutes in a substance-specific manner (**Figure 4**). Soluble sugar content peaked at T2, the highest among all treatments, partly restoring the sugar level toward that of CK, although this increase did not reach statistical significance. The soluble protein content did not differ significantly from CK under T1 and T3, was restored to the CK level under T2, and was comparable to LT under T4; it was significantly lower than in CK only under T5. Glycine betaine responded weakly: only T1 produced a slight (non-significant) increase, and the higher concentrations reduced its accumulation further, with the contents under T2, T4 and T5 significantly lower than in LT (p < 0.05). Proline was the most responsive of the four solutes: on top of its cold-induced accumulation, trehalose pretreatment further raised the proline content as the concentration increased, peaking at T2 (+40.3% vs. LT; p < 0.05) and then declining, falling significantly below the LT level at the highest concentration (T5). Because soluble sugars and proline both peaked at T2, the two solutes appear to contribute jointly to the osmotic adjustment promoted by trehalose.

These results suggest that exogenous trehalose improves osmotic adjustment in grape seedlings under low-temperature stress mainly by promoting the accumulation of soluble sugars and free proline. In addition to lowering cellular osmotic potential, these solutes stabilize membranes and proteins, which may be one route by which trehalose relieves cold stress in grape.

### 3.4 Maintenance effects of exogenous trehalose on ion homeostasis under low-temperature stress

The maintenance of intracellular ion homeostasis underlies normal physiological metabolism. Low-temperature stress disrupts the selective permeability of cell membranes, leading to Na^+^ influx and K^+^ efflux and thus to ion imbalance and metabolic disorder. As shown in **Figure 5**, compared with CK, 0 °C low-temperature stress significantly increased the Na^+^/K^+^ ratio in grape leaves (p < 0.05); the Na^+^ content increased numerically by 44.9%, whereas the 10.6% decrease in K^+^ content was not statistically significant, together indicating severe ion imbalance under low-temperature stress.

**Figure 5:**
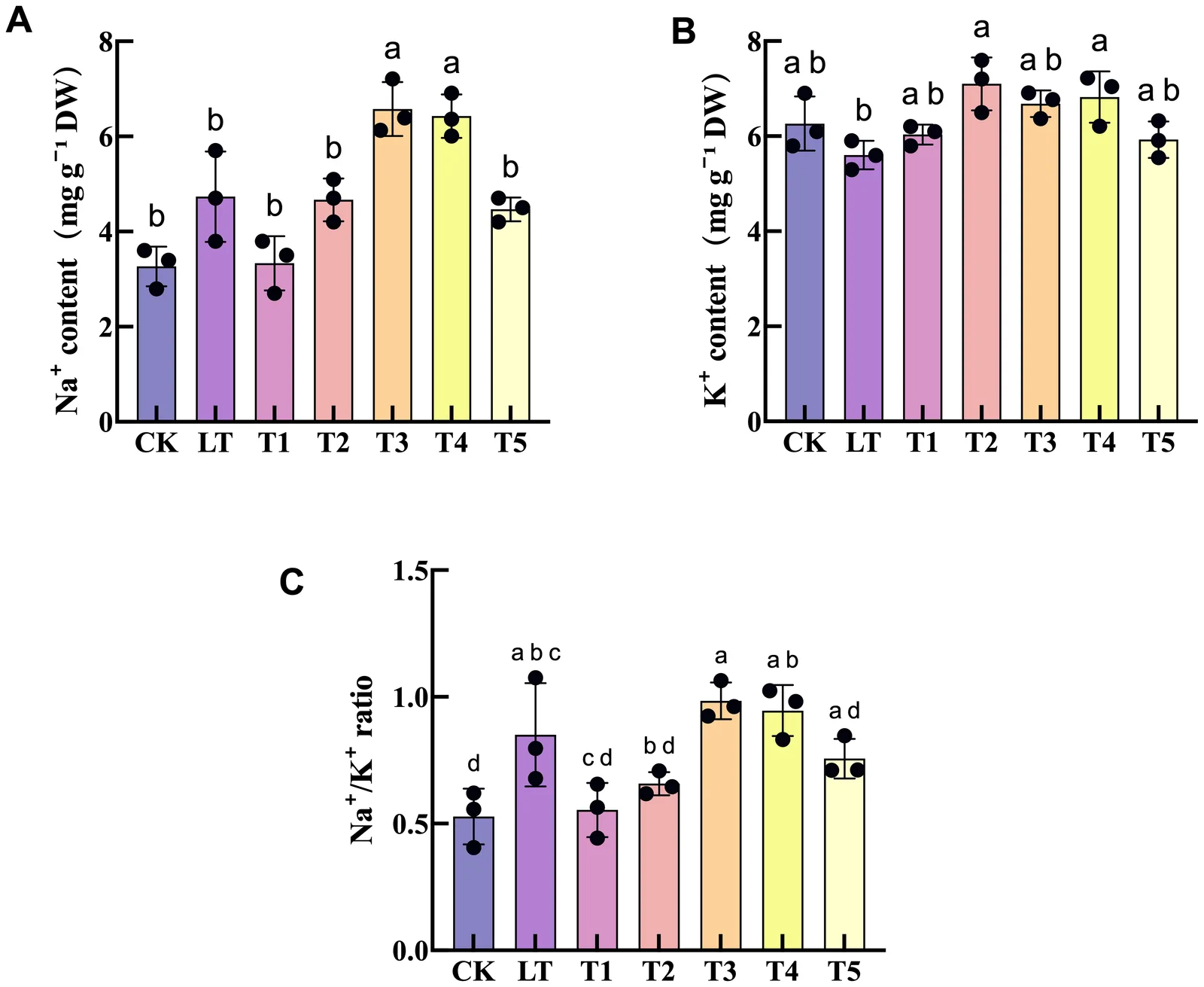
Effects of different concentrations of exogenous trehalose on ion homeostasis in ‘Kyoho’ grape leaves under low-temperature stress. **(A)** Sodium ion (Na^+^, mg g^−1^ DW). **(B)** Potassium ion (K^+^, mg g^−1^ DW). **(C)** Na^+^/K^+^ ratio. Different lowercase letters indicate significant differences among treatments (Duncan’s multiple range test, p < 0.05). Error bars represent the mean ± standard deviation (Mean ± SD, n = 3).

Pretreatment with exogenous trehalose regulated ion homeostasis in a concentration-dependent manner rather than following a simple “higher concentration is better” pattern. T1 (50 mmol L^−1^) showed the best regulation: the Na^+^/K^+^ ratio decreased by 34.8% compared with LT, the lowest among all treatments, followed by T2. Specifically, T1 reduced the Na^+^ content numerically, although this difference was not statistically significant, whereas T2 had no significant effect on Na^+^ but significantly promoted K^+^ uptake: the K^+^ content under T2 increased by 26.8% relative to LT (p < 0.05), whereas the 19.3% increase under T3 was not statistically significant. In contrast, the Na^+^ contents under T3 and T4 were significantly higher than under LT (p < 0.05), and their Na^+^/K^+^ ratios (0.98 and 0.95, respectively) also numerically exceeded the LT level, indicating that 150–200 mmol L^−1^ did not improve ion homeostasis and tended to aggravate ion imbalance. T5 reduced the Na^+^/K^+^ ratio by 11.0% relative to LT, an effect weaker than those of T1 and T2. These results indicate that low concentrations of exogenous trehalose, particularly 50–100 mmol L^−1^, help maintain the selective permeability of the cell membrane, limit intracellular Na^+^ accumulation and promote K^+^ uptake, thereby contributing to intracellular ion homeostasis under low-temperature stress. Because only ion contents, rather than the activities of specific ion transporters, were measured, the transport pathways involved remain to be investigated.

### 3.5 Regulatory effects of exogenous trehalose on energy and respiratory metabolism under low-temperature stress

Energy and respiratory metabolism are central to plant responses to abiotic stress, and the functional stability of the mitochondrial respiratory chain directly determines the cellular energy supply and redox homeostasis. As shown in **Figure 6**, compared with CK, 0 °C low-temperature stress significantly inhibited the activity of cytochrome c oxidase (CCO), a key mitochondrial respiratory enzyme, and lowered the respiration rate in grape leaves (p < 0.05). Meanwhile, the content of the pyridine nucleotide NAD(H) decreased numerically by 18.8%, whereas NADP(H) decreased by only 13.6%, although neither decrease was statistically significant. Together, these results indicate that low-temperature stress disrupted the stability of the mitochondrial respiratory chain, suppressed aerobic respiration, and disturbed cellular energy status and redox homeostasis.

**Figure 6:**
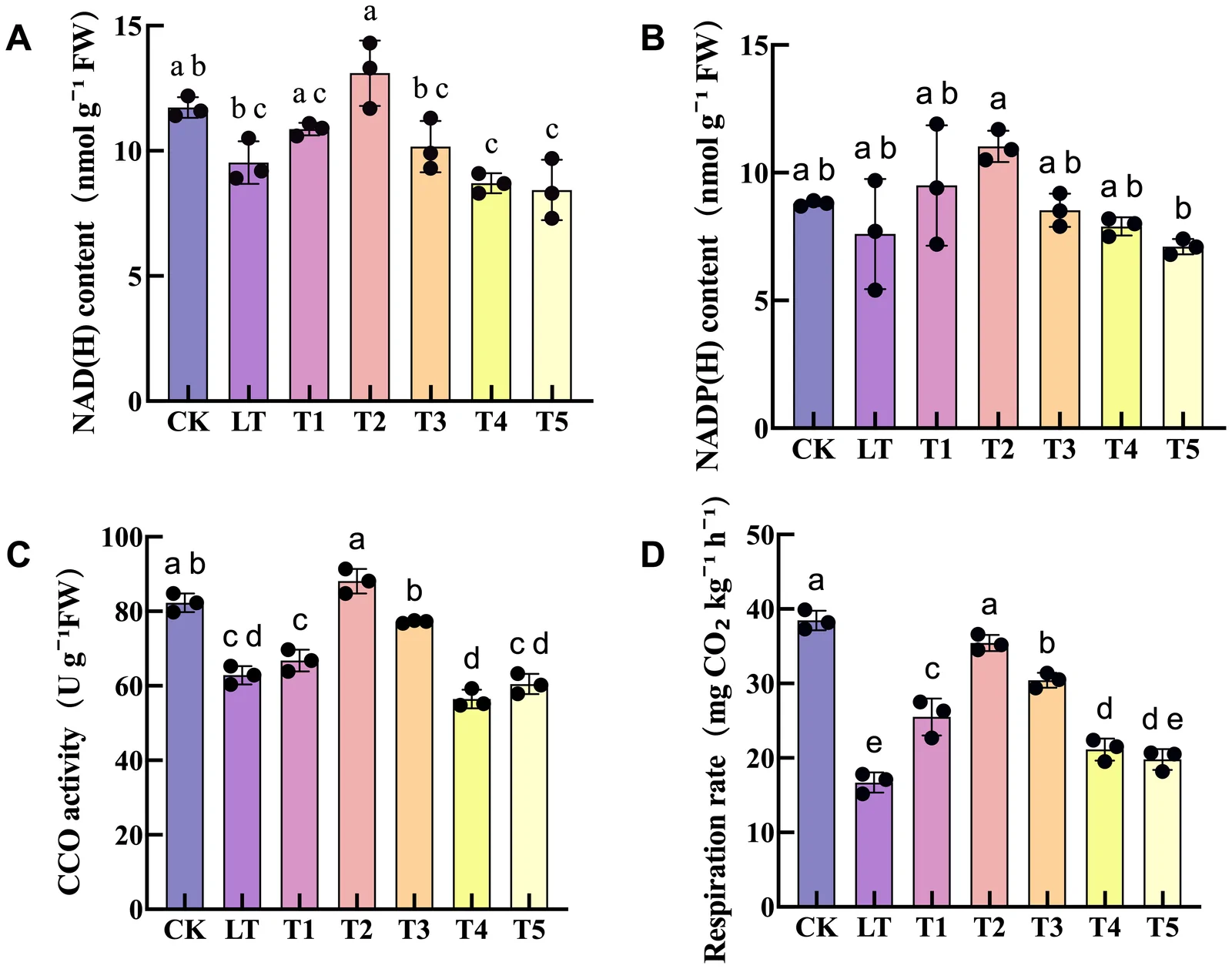
Effects of different concentrations of exogenous trehalose on key indicators of energy-respiratory metabolism in ‘Kyoho’ grape leaves under low-temperature stress. **(A)** Nicotinamide adenine dinucleotide (NAD(H), nmol g⁻¹ FW). **(B)** Nicotinamide adenine dinucleotide phosphate (NADP(H), nmol g⁻¹ FW). **(C)** Cytochrome c oxidase (CCO, U g^−1^ FW). **(D)** Respiration rate (RR, mg CO_2_ kg^−1^ FW h^−1^). Different lowercase letters indicate significant differences among treatments (Duncan’s multiple range test, p < 0.05). Error bars represent the mean ± standard deviation (Mean ± SD, n = 3).

Pretreatment with exogenous trehalose significantly activated key respiratory enzymes and increased the contents of reduced pyridine nucleotides in a concentration-dependent manner. T2 showed the optimal regulation: CCO activity and NAD(H) content increased by 40.1% and 37.4%, respectively, compared with LT (p < 0.05), and NADP(H) showed the largest relative increase (+45.2%), although this difference was not statistically significant. These results suggest that appropriate concentrations of exogenous trehalose help preserve mitochondrial respiratory-chain function by enhancing CCO activity, thereby supporting aerobic respiration and energy supply under low-temperature stress. In addition, the trehalose-induced increase in NADP(H) may provide reducing equivalents for ROS-scavenging pathways such as the AsA-GSH cycle, pointing to a potential link between energy-respiratory metabolism and antioxidant defense. As these inferences rest on correlational, activity-level measurements, the causal cascade linking CCO regulation to antioxidant defense awaits verification through gene-expression, protein-modification or genetic evidence.

### 3.6 Correlation analysis of physiological indicators

Pearson correlation analysis was performed across all measured physiological indicators; the representative correlation coefficients are presented in **Table 2**. Membrane system damage and ROS burst were tightly coupled: REC, H_2_O_2_ and O_2_·^−^ were highly significantly positively correlated (r = 0.800–0.877, p < 0.001), with the strongest associations observed between REC and O_2_·^−^ (r = 0.877) and between H_2_O_2_ and O_2_·^−^ (r = 0.876). MDA was also positively correlated with H_2_O_2_ (r = 0.651, p < 0.01), whereas its correlations with REC and O_2_·^−^ were weaker and non-significant. This indicates that, under low-temperature stress, the excessive production of O_2_·^−^ is tightly associated with the loss of membrane integrity (REC) and with H_2_O_2_ accumulation, and that the coupled amplification of ROS burst and membrane permeability constitutes the initial core link of low-temperature injury in ‘Kyoho’ grapes.

**Table 2:** Pearson correlation matrix among the measured physiological indicators in ‘Kyoho’ grape leaves under low-temperature stress treated with different concentrations of exogenous trehalose (n = 21).

Indicator	Na^+^	K^+^	SS	SP	Pro	GB	AsA	GSH	TP	FLA	REC	MDA	H_2_O_2_	O_2_·^−^	NAD(H)	NADP(H)	CCO	RR	SOD	POD	CAT
Na^+^	1.000																				
K^+^	0.338	1.000																			
SS	-0.440*	0.249	1.000																		
SP	-0.200	0.337	0.554**	1.000																	
Pro	0.420	0.301	-0.624**	-0.363	1.000																
GB	-0.423	-0.154	0.363	0.378	-0.430	1.000															
AsA	-0.165	0.214	0.564**	0.623**	-0.326	0.453*	1.000														
GSH	-0.173	0.494*	0.658**	0.336	-0.261	0.285	0.735***	1.000													
TP	-0.273	0.327	0.427	0.379	-0.008	0.451*	0.698***	0.697***	1.000												
FLA	-0.285	-0.212	0.350	0.274	-0.671***	-0.127	0.163	0.166	-0.087	1.000											
REC	0.438*	-0.312	-0.651**	-0.443*	0.493*	-0.533*	-0.517*	-0.801***	-0.530*	-0.330	1.000										
MDA	-0.279	-0.658**	-0.235	0.148	-0.174	0.397	-0.223	-0.677***	-0.320	-0.045	0.301	1.000									
H_2_O_2_	0.301	-0.415	-0.677***	-0.193	0.286	-0.315	-0.614**	-0.935***	-0.661**	-0.113	0.800***	0.651**	1.000								
O_2_·^−^	0.529*	-0.221	-0.652**	-0.353	0.391	-0.640**	-0.682***	-0.857***	-0.723***	-0.126	0.877***	0.306	0.876***	1.000							
NAD(H)	-0.312	0.352	0.399	0.480*	0.020	0.451*	0.680***	0.644**	0.707***	0.024	-0.614**	-0.182	-0.576**	-0.744***	1.000						
NADP(H)	-0.124	0.348	0.187	0.441*	0.272	0.281	0.560**	0.499*	0.657**	-0.211	-0.376	-0.228	-0.436*	-0.494*	0.714***	1.000					
CCO	-0.204	0.424	0.544*	0.452*	-0.090	0.455*	0.855***	0.816***	0.834***	0.006	-0.596**	-0.375	-0.729***	-0.793***	0.855***	0.574**	1.000				
RR	-0.227	0.432	0.626**	0.402	-0.260	0.488*	0.786***	0.950***	0.777***	0.049	-0.801***	-0.509*	-0.912***	-0.916***	0.730***	0.591**	0.867***	1.000			
SOD	-0.359	0.149	0.651**	0.600**	-0.549*	0.748***	0.782***	0.738***	0.634**	0.240	-0.820***	-0.047	-0.661**	-0.860***	0.727***	0.439*	0.775***	0.841***	1.000		
POD	-0.199	0.344	0.690***	0.433*	-0.634**	0.368	0.593**	0.831***	0.500*	0.472*	-0.875***	-0.459*	-0.807***	-0.756***	0.425	0.218	0.570**	0.806***	0.777***	1.000	
CAT	-0.236	0.403	0.696***	0.599**	-0.439*	0.507*	0.760***	0.861***	0.673***	0.286	-0.873***	-0.324	-0.788***	-0.841***	0.733***	0.442*	0.811***	0.901***	0.918***	0.886***	1.000

*p < 0.05; **p < 0.01; ***p < 0.001 (two-tailed).

The antioxidant system presented a highly synergistic regulatory mode. Within the enzymatic arm, SOD, POD and CAT were highly significantly positively correlated (r = 0.775–0.918, p < 0.001). Within the non-enzymatic arm, AsA, GSH and total phenols were likewise tightly coupled (r = 0.697-0.735, p < 0.001), supporting the operation of the AsA-GSH cycle; by contrast, flavonoids were only weakly and mostly non-significantly correlated with the other antioxidant indicators (except for a moderate correlation with POD). Across the two arms, the strongest association was between CAT and GSH (r = 0.861, p < 0.001). At the same time, GSH and the major antioxidant enzymes were highly significantly negatively correlated with the damage variables, with r values as low as -0.935 (GSH vs. H_2_O_2_), -0.860 (SOD vs. O_2_·^−^) and -0.857 (GSH vs. O_2_·^−^), although flavonoids showed no significant correlation with any damage indicator. This pattern is consistent with the view that trehalose restricts excessive ROS accumulation and alleviates membrane oxidative damage by coordinately activating the enzymatic and AsA-GSH non-enzymatic antioxidant systems.

Within the osmotic-adjustment system, the stress-resistant contributions differed markedly among substances. Soluble sugars were significantly negatively correlated with REC, H_2_O_2_ and O_2_·^−^ (r = −0.651, −0.677 and −0.652, respectively; p < 0.01, p < 0.001 and p < 0.01) and significantly positively correlated with GSH and with SOD, POD and CAT, consistent with a central role for soluble sugars in linking trehalose to improved antioxidant capacity. Soluble proteins showed only weak-to-moderate associations with the damage variables, and glycine betaine, although it correlated positively with SOD (r = 0.748), was not significantly increased by trehalose (see Section 3.3), indicating that both play an auxiliary rather than central role in the trehalose stress-resistant network.

Ion-homeostasis imbalance was associated with low-temperature injury, although more weakly than the membrane/ROS module itself. Across all samples, leaf Na^+^ content was significantly positively correlated with REC (r = 0.438) and O_2_·^−^ (r = 0.529), whereas K^+^ content was most strongly and negatively correlated with MDA (r = -0.658, p < 0.01); the correlations of K^+^ with REC, H_2_O_2_ and O_2_·^−^ were weaker and non-significant. Together with the treatment-level evidence in 3.4, this indicates that appropriate trehalose concentrations (50–100 mmol L^−1^) help preserve membrane selective permeability, restrict Na^+^ accumulation and favor K^+^ retention, thereby contributing to intracellular ion homeostasis and indirectly mitigating low-temperature secondary damage.

Of particular note, the indicators of the energy-respiratory metabolism module were strongly coupled. CCO, NAD(H), NADP(H) and respiration rate (RR) were significantly positively inter-correlated (r = 0.574–0.867, p < 0.01 or p < 0.001), with the strongest associations between CCO and RR (r = 0.867) and between CCO and NAD(H) (r = 0.855). This module also showed the strongest negative coupling to ROS: RR was highly significantly negatively correlated with H_2_O_2_ and O_2_·^−^ (r = -0.912 and -0.916, p < 0.001), and CCO with O_2_·^−^ (r = -0.793); NADP(H), by contrast, was only weakly correlated with the damage variables. At the same time, the energy-respiratory indicators were strongly and positively coupled to the antioxidant system, most notably RR with GSH (r = 0.950) and with CAT (r = 0.901), and CCO with AsA (r = 0.855) and GSH (r = 0.816). This supports the notion that energy-respiratory metabolism acts as a core hub through which exogenous trehalose regulates low-temperature resistance in ‘Kyoho’ grapes: stabilizing the mitochondrial respiratory chain (CCO) to secure energy supply and maintaining pyridine-nucleotide reducing power to feed ROS-scavenging pathways such as the AsA-GSH cycle, thereby achieving coordinated regulation of energy metabolism and antioxidant defense.

### 3.7 Principal component analysis of physiological indicators

To further visualize the overall separation among treatments and the contribution of individual indicators to this separation, a principal component analysis (PCA) was performed on all measured physiological parameters (**Figure 7**). The first two principal components collectively explained 68.9% of the total variance (PC1 = 54.3%, PC2 = 14.6%). Along PC1, which represented the axis of oxidative damage versus antioxidant protection, the low-temperature stress control (LT) was clearly separated from the unstressed control (CK): LT was clustered on the negative-PC1 side, closely associated with damage-related variables including REC, H_2_O_2_ and O_2_·^−^, whereas CK was shifted toward the positive-PC1 side, associated with protective variables such as soluble sugar, AsA, GSH, total phenols, NADP(H), K^+^ and the energy-respiratory indicators. Among the trehalose treatments, T2 was positioned closest to CK and well separated from LT, indicating that this concentration most effectively restored the overall physiological profile toward the unstressed state. T1 and T3 showed intermediate positions between T2 and LT, while the high-concentration treatments T4 and T5 shifted back toward the LT side, confirming the concentration-dependent decline in protective effects at supra-optimal doses. Along PC2, proline, K^+^ and Na^+^ loaded most strongly in the positive direction, whereas MDA, flavonoids and glycine betaine loaded in the negative direction, reflecting the distinct osmotic-adjustment and ion-homeostasis axes of the stress response. These PCA results are consistent with the correlation analysis (**Table 2**) and provide an integrated, multivariate confirmation that 100 mmol L^−1^ is the optimal trehalose concentration for alleviating low-temperature injury in ‘Kyoho’ grape.

**Figure 7:**
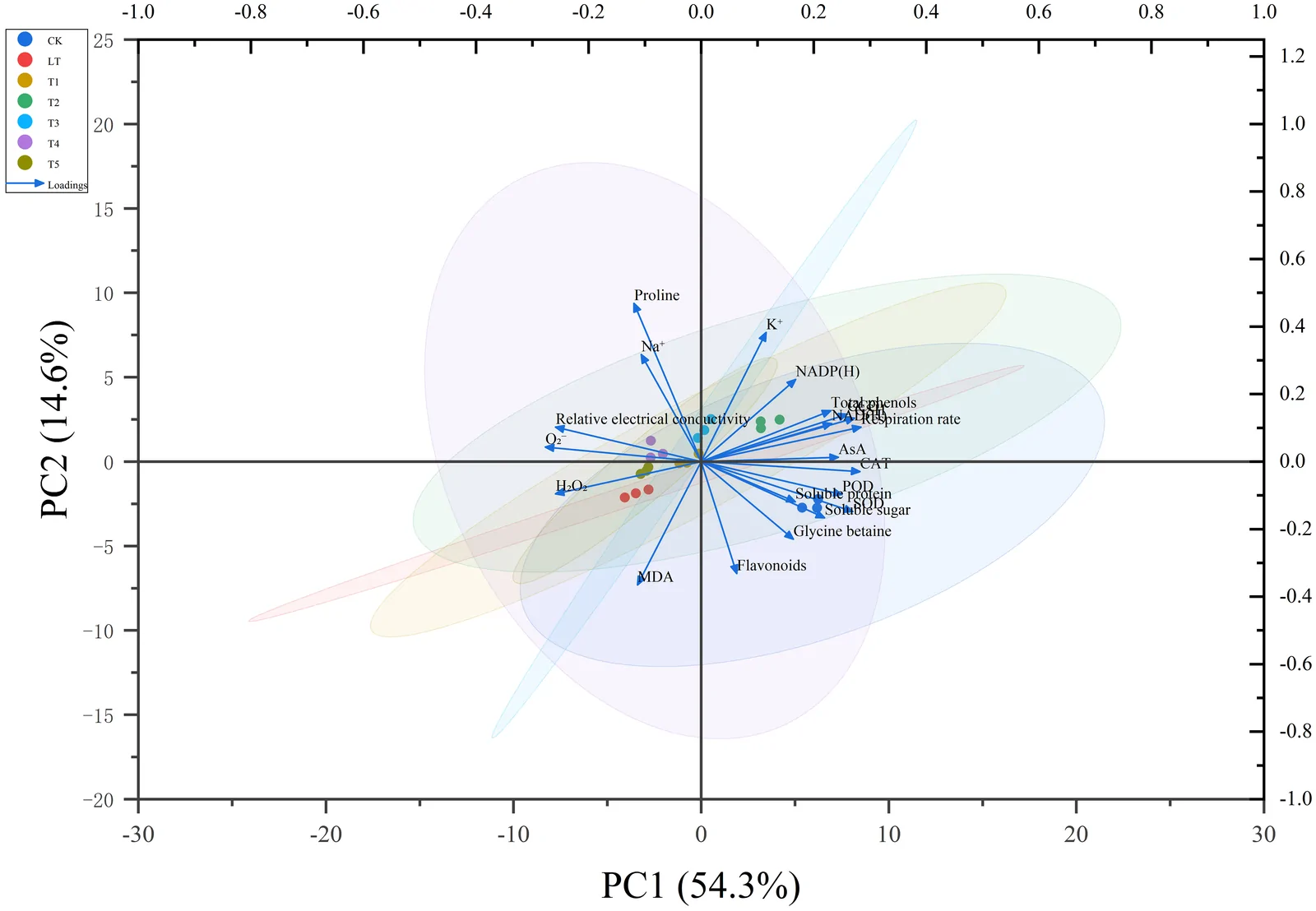
Principal component analysis (PCA) biplot of physiological indicators in ‘Kyoho’ grape leaves under low-temperature stress treated with different concentrations of exogenous trehalose. PC1 and PC2 explained 54.3% and 14.6% of the total variance, respectively. Different colors represent the seven treatment groups (CK, LT, T1-T5); shaded ellipses indicate 95% confidence intervals for each group. Blue arrows represent the loading vectors of individual physiological parameters (MDA, H_2_O_2_, O_2_·^−^, REC, SOD, POD, CAT, AsA, GSH, total phenols, flavonoids, soluble sugar, soluble protein, proline, glycine betaine, Na^+^, K^+^, CCO, NAD(H), NADP(H), and respiration rate).

## 4 Discussion

In this study, foliar trehalose pretreatment mitigated low-temperature injury in ‘Kyoho’ grape seedlings in a concentration-dependent manner, with 100 mmol L^−1^ identified as the optimal concentration, and provided correlational evidence that the coordinated regulation of energy-respiratory metabolism and antioxidant defense constitutes an important physiological mechanism of this protection. These results refine the physiological framework of grape cold resistance and provide a quantitative basis for chemical regulation in grape-producing regions at risk of low temperature.

### 4.1 Concentration-dependent effects of exogenous trehalose on cold resistance in grape

The alleviating effect of exogenous trehalose on low-temperature stress in ‘Kyoho’ grape was strongly concentration-dependent, with 100 mmol L^−1^ being the optimal concentration. Concentrations below or above this optimum reduced the protective effect, which was largely lost at high concentrations. This “low promotion and high inhibition” concentration-dependent effect is consistent with the enhanced stress tolerance associated with trehalose metabolism in rice (Li et al., 2011) and with the effects of exogenous trehalose application in watermelon (Yuan et al., 2022), indicating that such a dose-response pattern may be a common characteristic of trehalose-mediated regulation of abiotic stress responses in plants. Such a hormesis-like, low-promotion/high-inhibition dose-response has likewise been reported for other exogenous protective regulators (e.g., salicylic acid, betaine and nitric oxide donors) under low temperature and other abiotic stresses, where supra-optimal concentrations become phytotoxic (Afzal et al., 2021; Pan et al., 2023). The PCA biplot further supported this concentration-dependent pattern: T2 occupied the position closest to CK, whereas T4 and T5 reverted toward the LT cluster on the first principal component, providing multivariate evidence that the protective effect of trehalose is largely lost at concentrations ≥200 mmol L^−1^.

Pan et al. (2023) preliminarily explored the concentration range (50–200 mmol L^−1^) of exogenous trehalose for regulating the salt-stress response of tomato, but precise concentration thresholds under low-temperature stress, particularly in grape, remain undefined. By establishing a concentration gradient of 50–250 mmol L^−1^, the present study determined the optimal foliar application concentration of trehalose for ‘Kyoho’ grape under 0 °C stress to be 100 mmol L^−1^, providing a directly referable quantitative parameter for the practical use of chemical regulation in cold-resistant grape cultivation.

### 4.2 Exogenous trehalose alleviates low-temperature oxidative damage in grape

Membrane lipid peroxidation triggered by excessive ROS accumulation is a central event in low-temperature injury of grapevine (Zhang et al., 2023). This study found that 100 mmol L^−1^ exogenous trehalose significantly reduced REC and MDA accumulation in grape leaves, reduced the excessive accumulation of H_2_O_2_ and O_2_·^−^, and synergistically activated antioxidant enzymes such as SOD, CAT and POD while promoting the accumulation of non-enzymatic antioxidants such as AsA, GSH and total phenols. This result is consistent with the research conclusions of Shi et al. (2019) in tobacco, of Feng et al. (2019) in tomato and of Wang et al. (2024) in peach fruit, supporting the view that trehalose accelerates the removal of excess intracellular ROS by simultaneously strengthening the enzymatic and non-enzymatic antioxidant defense systems, thereby alleviating low-temperature-induced oxidative damage. At the mechanistic level, trehalose-induced cold tolerance has been linked to H_2_O_2_ signaling and aquaporin-facilitated antioxidative responses in melon seedlings (Han et al., 2024).

Among the non-enzymatic antioxidants, GSH showed the strongest response to trehalose, and its content under the T2 treatment was significantly higher than in the LT group (p < 0.05). This finding agrees with the central role of glutathione in plant ROS scavenging proposed by Foyer and Halliwell (1976) and suggests that the AsA-GSH cycle is the core non-enzymatic pathway through which trehalose regulates antioxidant defense under low temperature in grape. As a key component of the AsA-GSH cycle, GSH can not only directly scavenge ROS, but also regenerate AsA by reducing dehydroascorbic acid (DHA) to maintain the reduced state of the AsA pool (Rahman et al., 2006). The marked increase in GSH may therefore be an important basis for the efficient ROS scavenging and alleviation of oxidative damage observed under trehalose pretreatment.

### 4.3 Synergistic regulation of energy-respiratory metabolism and antioxidant defense

A major finding of this study is the correlational evidence that the synergistic regulation of energy-respiratory metabolism and the antioxidant defense system is a central physiological mechanism by which exogenous trehalose alleviates low-temperature stress in grape. The mitochondrial respiratory chain is the main site of cellular energy synthesis in plants, and its functional stability directly determines the cellular energy supply. As a key enzyme of mitochondrial respiratory-chain complex IV, CCO activity directly reflects the functional state of the respiratory chain (Ribas-Carbo et al., 1994). In this study, under T2, CCO activity was significantly higher than that in the LT group (p < 0.05), indicating that trehalose can sustain aerobic respiration under low temperature by stabilizing mitochondrial respiratory-chain complex IV and thereby support the energy supply required for stress-resistance processes.

Meanwhile, trehalose increased the contents of both NAD(H) and NADP(H), with the relative increase being larger for NADP(H). NADPH is the principal source of cellular reducing power (Gibon and Larher, 1997). In the AsA-GSH cycle, glutathione reductase consumes NADPH to regenerate reduced glutathione (GSH) from its oxidized form (GSSG), thereby sustaining ROS scavenging (Foyer and Halliwell, 1976; Nakano and Asada, 1981). Therefore, the observed increase in NADP(H) content is consistent with the possibility that trehalose supplies reducing equivalents to the AsA-GSH cycle and supports its efficient operation.

Based on the present correlational and activity-level evidence, we propose a working model in which trehalose may contribute to the synergistic regulation of energy-respiratory metabolism and antioxidant defense through a pathway involving enhanced CCO activity supporting energy supply and a strengthened AsA-GSH cycle scavenging ROS. This working model is consistent with the view that mitochondrial respiratory-chain stability is central to plant stress resistance, extends the findings of Yu et al. (2021) on energy metabolism during the grapevine stress response, and agrees with recent multi-omics evidence that exogenous trehalose enhances cold tolerance in grape (Wang et al., 2026). However, the causal nature of this cascade model requires further validation through multi-omics and molecular approaches. In particular, future work will use RT-qPCR to examine Vitis orthologs of representative stress-responsive genes across treatments, including the cold-signaling CBF1/CBF2 (DREB1) family and their downstream COR15A marker, the proline-biosynthesis genes P5CS1 and P5CR, and the redox-homeostasis genes GR1, GR2 and DHAR; this will test whether the physiological protection conferred by trehalose is accompanied by coordinated transcriptional activation of cold- and oxidative-stress pathways.

### 4.4 Auxiliary roles of osmotic adjustment and ion homeostasis

In addition to these core mechanisms, exogenous trehalose improved the osmoregulatory capacity of grape seedlings through the marked accumulation of soluble sugars and proline. Soluble sugars function as osmotic regulators that lower cellular osmotic potential and limit low-temperature-induced cellular dehydration, and they also protect macromolecules by forming hydrogen bonds with proteins and phospholipids (Takahashi and Murata, 2008). As a compatible osmolyte and stress signal, proline accumulates under cold stress, stabilizes proteins and membranes, and cooperates with the antioxidant system by scavenging hydroxyl radicals (Bates et al., 1973). Trehalose itself is also an effective osmoprotectant and macromolecule stabilizer, and foliar-applied trehalose can be taken up by leaf cells, which may further contribute to the observed protection.

Meanwhile, appropriate concentrations of trehalose may also preserve the selective permeability of the cell membrane, modulate ion transport processes, limit intracellular Na^+^ accumulation and promote K^+^ uptake, thereby helping to maintain intracellular Na^+^/K^+^ homeostasis. This result is consistent with the mechanism by which exogenous regulators maintain ion homeostasis, as reported by Afzal et al. (2021) in wheat, and complements the proposed regulatory network of trehalose-mediated stress resistance.

### 4.5 Limitations and future perspectives

This study was conducted with potted one-year-old ‘Kyoho’ grape cutting seedlings grown indoors under a single, artificially controlled low-temperature stress, and did not consider the combined effects of low temperature with drought, high light or other stresses under field conditions, nor did it compare responses among growth stages (Aran et al., 2017; Amulya et al., 2024). The field applicability of these results therefore requires further validation. Future work should include multi-year, multi-location field trials to define the optimal trehalose concentration, application period and frequency for different ecological regions and growth stages (Schultz, 2000). Combining transcriptomic, metabolomic and proteomic analyses with targeted RT-qPCR of the cold- and oxidative-stress marker genes noted above should help clarify the molecular mechanism of trehalose-induced cold resistance in grape and identify key regulatory genes for molecular breeding. In addition, the combined application of trehalose with other stress-resistance agents, such as salicylic acid and abscisic acid, warrants exploration to further improve cold protection and to provide more comprehensive technical support for high-quality grape production.

## 5 Conclusion

In this study, foliar pretreatment with exogenous trehalose mitigated low-temperature (0 °C) injury in one-year-old ‘Kyoho’ grape cutting seedlings in a clear concentration-dependent manner. The optimal concentration was 100 mmol L^−1^ (T2), which significantly reduced membrane lipid peroxidation and ROS accumulation, enhanced both the enzymatic (SOD, POD and CAT) and the non-enzymatic (AsA, GSH and total phenols) antioxidant defenses, stimulated energy-respiratory metabolism (CCO, NAD(H), NADP(H) and respiration rate), and helped preserve osmotic adjustment and ion homeostasis. Principal component analysis further showed that T2 was clearly separated from LT and clustered closest to CK along PC1 (54.3% of the total variance), indicating that this concentration best restored the overall physiological profile toward the unstressed state. These findings provide a directly usable quantitative parameter for chemical regulation and a physiological framework for cold-resistant grape cultivation in low-temperature-risk regions.

## Data Availability

The original contributions presented in the study are included in the article/**Supplementary Material**, further inquiries can be directed to the corresponding author/s.

## Supplementary material

Supplementary Figure S1 One-year-old ‘Kyoho’ grape cutting seedlings used as the experimental material.

Supplementary Figure S2 Schematic diagram of the experimental procedure, including seedling acclimation, foliar trehalose spraying, 0 °C lowtemperature treatment for 7 d, and sampling.
